# Benefits and concerns of probiotics: an overview of the potential genotoxicity of the colibactin-producing *Escherichia coli* Nissle 1917 strain

**DOI:** 10.1080/19490976.2024.2397874

**Published:** 2024-09-04

**Authors:** Luca Falzone, Alessandro Lavoro, Saverio Candido, Mario Salmeri, Antonino Zanghì, Massimo Libra

**Affiliations:** a Department of Biomedical and Biotechnological Sciences, University of Catania, Catania, Italy; b Research Center for Prevention, Diagnosis and Treatment of Cancer, University of Catania, Catania, Italy; c Department of Medical and Surgical Sciences and Advanced Technology ‘G.F. Ingrassia’, University of Catania, Catania, Italy

**Keywords:** Gut microbiota, Probiotics, Postbiotics, probiotic safety, *Escherichia coli* Nissle 1917, *EcN*, Colibactin, *pks* locus, Genotoxicity, CRC

## Abstract

Recently, the mounting integration of probiotics into human health strategies has gathered considerable attention. Although the benefits of probiotics have been widely recognized in patients with gastrointestinal disorders, immune system modulation, and chronic-degenerative diseases, there is a growing need to evaluate their potential risks. In this context, new concerns have arisen regarding the safety of probiotics as some strains may have adverse effects in humans. Among these strains, *Escherichia coli* Nissle 1917 (*EcN*) exhibited traits of concern due to a pathogenic locus in its genome that produces potentially genotoxic metabolites. As the use of probiotics for therapeutic purposes is increasing, the effects of potentially harmful probiotics must be carefully evaluated. To this end, in this narrative review article, we reported the findings of the most relevant *in vitro* and *in vivo* studies investigating the expanding applications of probiotics and their impact on human well-being addressing concerns arising from the presence of antibiotic resistance and pathogenic elements, with a focus on the polyketide synthase (*pks*) pathogenic island of *EcN*. In this context, the literature data here discussed encourages a thorough profiling of probiotics to identify potential harmful elements as done for *EcN* where potential genotoxic effects of colibactin, a secondary metabolite, were observed. Specifically, while some studies suggest *EcN* is safe for gastrointestinal health, conflicting findings highlight the need for further research to clarify its safety and optimize its use in therapy. Overall, the data here presented suggest that a comprehensive assessment of the evolving landscape of probiotics is essential to make evidence-based decisions and ensure their correct use in humans.

## Introduction

The human microbiota, a diverse community of microorganisms residing in different sites of the human body, plays a central role in maintaining human health. This complex ecosystem influences various physiological processes, including digestion, immune function, metabolism, and even neurological health.^1^ The composition and diversity of the microbiota have been linked to numerous disorders ranging from gastrointestinal diseases to obesity and mental health issues.^2^ Consequently, understanding the factors influencing microbiota composition and promoting a balanced microbiota is critical to well-being.^3^

In this context, several studies have shown the beneficial effects of administering specific microorganisms that promote microbiota homeostasis and, consequently, human health.^4,5^ Such microorganisms are commonly referred to as probiotics, which are live bacteria or yeasts that, when administered in sufficient quantities, provide health benefits to the host. Probiotics are commonly found in certain foods and dietary supplements and include a variety of bacterial strains, such as *Lacticaseibacillus*, *Lactobacillus,* and *Bifidobacterium* genera.^6^ Probiotics can improve the diversity and stability of gut microbiota, contributing to host homeostasis. These beneficial effects are mainly achieved by outcompeting harmful bacteria, creating a more favorable environment for beneficial species, and influencing the gut’s immune response.^7,8^

As living microorganisms, probiotics metabolize and produce substances that help the host in detoxifying harmful compounds or counteract pathogen adhesion. The products of probiotic metabolism are defined as postbiotics and include all bioactive compounds with a biological function. These compounds include short-chain fatty acids (SCFAs), peptides, enzymes, and various metabolites. Of note, postbiotics may contribute significantly to the health benefits attributed to probiotic consumption. They modulate immune responses, strengthen the intestinal epithelial barrier (IEB), and have anti-inflammatory and antioxidant effects.^9,10^ Although probiotics and postbiotics offer numerous health benefits, they can have potential adverse effects that may occur in certain situations.

Indeed, it has been reported that the administration of probiotics in immunocompromised individuals, or those with preexisting health conditions, can lead to infections. This is particularly true for people with weakened immune systems, such as pediatric patients, transplant recipients, or patients undergoing chemotherapy.^11^ In these cases, probiotics like some strains of *Lactocaseibacillus* spp, *Bifidobacteria* or *Bacillus subtilis* have been linked to infections, including severe sepsis.^12–14^ Less serious negative effects include bloating, indigestion, and gas production. However, these symptoms are generally transient and diminish when the gut microbiota adapts.^15^

In rare cases, the administration of probiotics can lead to hypersensitive reactions manifested by different symptoms, such as rashes, itching, and swelling.^16,17^ In addition, probiotics may alter the effects of concomitant treatments through interactions with the host, especially those based on the administration of immunosuppressive drugs or antibiotics, thus interfering with their efficacy.^18,19^ Despite these rare adverse effects, there is a general consensus on the safety of using well-known and widely tested probiotics such as *Lacticaseibacillus rhamnosus* GG, *Lactobacillus acidophilus*, *Ligilactobacillus salivarius*, *Saccharomyces boulardii*, *Bifidobacterium longum*, etc.^20–22^

Of note, postbiotics also showed negative effects on human health. In particular, some postbiotics may also modulate the immune system with unfavorable effects in individuals with autoimmune diseases by either enhancing or dampening immune responses.^23^ More importantly, bacteria and probiotics can be involved in the production of harmful metabolites and metabolism byproducts. A comprehensive metagenomic study has identified 117 histamine-secreting bacteria that are significantly enriched in inflammatory bowel diseases (IBD) patients with histamine-sensitivity inducing symptoms like headaches, itching, and digestive discomfort.^24–26^ In this context, certain probiotics can be involved in the production and metabolism of histamine reducing intestinal inflammation through the activation of the histamine H2 receptor.^27^

Other studies have shown that some probiotics produce genotoxic metabolites, like colibactin, or are associated with the transmission of antibiotic resistance elements.^28–30^

It is important to point out that most people can safely include probiotics and postbiotics into their diet without adverse effects. However, specific risks should be considered, especially for vulnerable populations, including individuals with weakened immune systems, preexisting health conditions, allergies, or young children.^31^

Despite their potential benefits, the safety of probiotics and postbiotics is a growing concern. In addition, probiotics are not classified as drugs by the U.S. Food and Drug Administration (FDA) and the European Medicines Agency (EMA), or other agencies.^32,33^ This classification raises challenges in evaluating and regulating the safety and efficacy of probiotics. Case reports of adverse events have highlighted the need for standardized testing and robust quality controls to ensure that probiotics are safe to consume, particularly in vulnerable populations. Therefore, the evolving landscape of probiotics underscores the need for rigorous safety assessments and regulatory frameworks to address their unique nature as living microorganisms rather than traditional pharmaceuticals.

To shed light on all these relevant aspects, in the present narrative review, a thorough search of the literature was performed utilizing various databases including PubMed/MedLine, Web of Science, Scopus, Google Scholar, and Cochrane Library. Specific keywords were employed to refine the search and select only relevant studies including “(probiotics) AND (probiotic safety) AND (genotoxicity)” and “(Escherichia coli Nissle 1917) AND (safety) AND (genotoxicity)” and other similar terms related to the main topic of the review.

Relevant studies on the role and safety of probiotics and the effects mediated by *Escherichia coli* Nissle 1917 (*EcN*) were selected. Priority was given to research articles with rigorous results and published in relevant journals. Articles that were not written in English, commentaries, editorials, and those without accessible full text, were excluded.

## Role of gut microbiota in human health and disease

Recent advances in metagenomics and high-throughput sequencing technologies (e.g. 16S rRNA Sequencing, Whole Genome Shotgun, or Whole RNA Shotgun) allowed the characterization of the gut microbiome, which refers to the whole genome from all microorganisms populating the gut microbiota.^34^ Conversely, gut microbiota consists of all the microbial communities living in the intestinal tract, of which bacteria represent the main community with more than 1,000 different bacterial species. In particular, *Firmicutes* and *Bacteroidetes* are the most abundant phyla, followed by *Actinobacteria*, *Fusobacteria*, *Proteobacteria*, and *Verrucomicrobia*. Besides bacteria, other commensal microorganisms may be identified in the human gut microbiota, especially yeasts, whose major types are represented by *Candida* and *Saccharomyces* genera, as well as viruses and archaea.^35,36^

Collectively, the gut microbiota is composed of trillions of microorganisms; however, the microbial composition within this environment is unique for each individual and may be affected by several factors. Age represents an important regulatory factor of the gut microbiota, whose diversity changes from childhood to adulthood. Specifically, it has been reported that the intraindividual variety and richness of gut microbes (α-diversity) increase with age, while interindividual differences relative to gut microbiota composition (β-diversity) decrease.^37,38^ Of note, recent studies highlighted that infant microbiota is initially defined by the mother, while the gut microbiota of three-year-old children overlaps with that of adults.^39,40^ Conversely, the elderly showed a significant reduction of *Bifidobacterium* and *Firmicutes*, whereas the relative abundance of *Enterobacteriaceae* and *Clostridia* increases triggering intestinal inflammation, which is strictly related to the onset of several pathological conditions.^41,42^

Gut microbiota is involved in several biological functions, including metabolism, nutrient absorption, synthesis of bioactive compounds, and fermentation.^43,44^ Moreover, gut microbiota prevents the colonization of harmful pathogens by competing for resources and producing antimicrobial compounds thus contributing to the maintenance of IEB integrity and functionality. Interestingly, the gut microbiota is also involved in immune system regulation, influencing its responsiveness to pathogens and contributing to immune homeostasis.^45,46^

**Figure 1.**
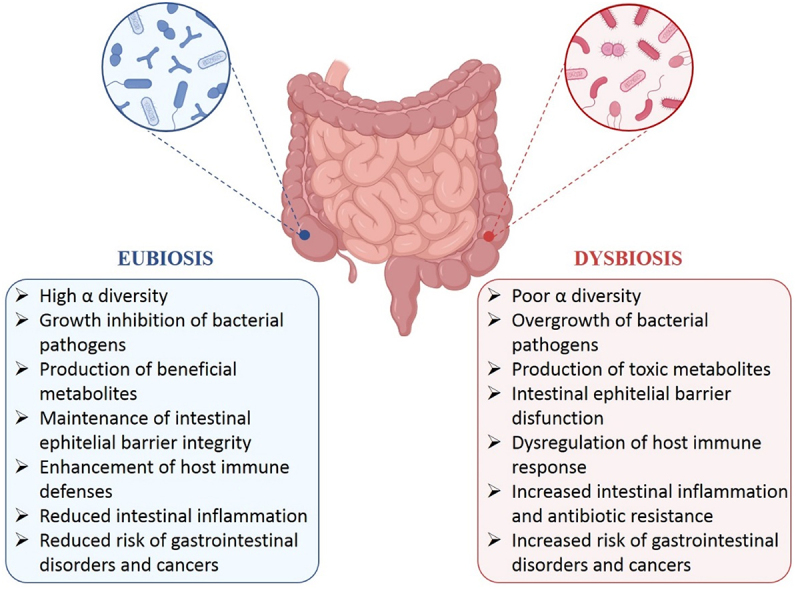
Main effects of gut microbiota eubiosis and dysbiosis.

Under physiological conditions, gut microbiota is characterized by a stable core microbiota, high α-diversity, and microbial gene richness (eubiosis) contributing to well-being. However, any imbalance in the composition and diversity of the microbial community within the gastrointestinal tract (dysbiosis) may lead to several pathological conditions, including obesity, diabetes, IBD, and cancer (Figure 1).^47,48^ Notably, gut dysbiosis is due to an overgrowth of pathogens compared to beneficial microbes, whose activity is related to genomic instability and tumor development. For instance, it has been reported that colibactin, a genotoxic secondary metabolite produced by different *Escherichia coli* strains, could play a critical role in colorectal cancer (CRC) initiation inducing DNA double-strand breaks (DSBs) in host cells and leading to genomic mutations.^49,50^ Similarly, other enteric pathogenic bacteria, such as *Shigella flexneri*, *Clostridioides difficile*, *Helicobacter pylori*, *Enterococcus faecalis*, *Fusobacterium nucleatum*, and *Bifidobacterium fragilis*, have been described in dysbiotic gut microbiota to release toxins triggering intestinal inflammation and disorders.^51–53^

In this context, microbiota-based therapy has recently emerged as an innovative approach to restore microbial eubiosis and maintain intestinal bacteria balance. Microbiota therapeutics include lifestyle modification, dietary interventions, prebiotics, probiotics, postbiotics, phage therapy, and fecal microbiota transplantation as main strategies for the management and treatment of various gastrointestinal disorders.^54–56^ Interestingly, it has been reported that this adjuvant therapeutic strategy allows the reduction of the severity and frequency of anti-cancer treatments and immunotherapy side effects, mainly diarrhea and mucositis.^45,57–59^ However, several challenges are associated with microbiota-based therapeutics and their effective application in clinical practice. Therefore, further studies are mandatory to understand the interplay between the gut microbiota community and the host.

## Probiotics in prevention and complementary therapy

As widely described in the previous subsection, human health is closely related to the composition of the gut microbiota.^36^ Among these microorganisms, a particular group is spotlighted for its potential to positively influence human health: probiotics. As widely known, probiotics are defined as living microorganisms that, when administered in appropriate amounts, provide health benefits to the host.^60,61^ These positive effects are achieved through some distinctive features that enable probiotics to contribute to human health. These include their ability to survive the acidic environment of the stomach (also defined as resilience), the immunomodulatory and anti-inflammatory effects, and the ability to adhere to the intestinal lumen and exert beneficial effects.^7^

In addition to these physical properties, probiotics influence the molecular and cellular functions of the surrounding cells and tissues through several mechanisms.^62^ Specifically, they compete with harmful microorganisms for resources through competitive exclusion, inhibit their growth, and decrease their gastrointestinal adhesion.^8^ Additionally, probiotics help strengthen IEB function and effectively prevent toxic substances or metabolites from entering the bloodstream.^63^

The term probiotics encompasses a variety of microorganisms, each with specific health-promoting properties. The first microorganisms to be recognized as probiotics were lactic acid bacteria (LAB), including well-known genera such as *Lacticaseibacillus* and *Streptococcus*.^64^ These bacteria, known for their ability to ferment sugars into lactic acid, are found in various fermented foods and dairy products and contribute to the maintenance of gastrointestinal homeostasis. Other known probiotics include the genera *Bifidobacterium* and *Enterobacterium*, which are known for their prevalence in the intestines of breastfed infants and their potential contribution to digestive health.^65,66^

Some probiotic strains can be introduced through specific foods, including breast milk or fermented foods (e.g. yogurt, kefir, sauerkraut, kimchi, and miso). In recent decades, probiotics have also been introduced into the human diet as supplements by encapsulating these living microorganisms.^67–69^

As keepers of normal gastrointestinal functions, probiotics strengthen the gut barrier by enhancing the integrity of tight junctions between gut epithelial cells.^70^ The disruption of IEB tightness, mediated by pathogenic bacteria, results in toxin infiltration and pro-inflammatory antigens into the extraintestinal tissues, determining different digestive tract disorders, systemic infections, autoimmune phenomena, and food intolerances.^71–73^ On the contrary, probiotics can promote the expression of proteins involved in the cell-cell interaction. In this context, zonulin was recently identified as a major component responsible for the regulation of intestinal cell tight junctions and its degradation was associated with a pathological condition defined as “leaky gut” or “leaky gut syndrome” characterized by chronic systemic inflammation and intestinal permeation. A growing body of research has also demonstrated the positive effects of integral gut microbiota and probiotics in inducing the expression and correct function of zonulin, suggesting how probiotics may promote health by regulating the homeostasis of the gut barrier.^63,74^

Closely connected to the integrity of the mucosa and IEB, there are also the effects of probiotics in favoring mucosal immunity. Indeed, probiotics orchestrate a variety of immune responses, modulating the activity of immune cells and influencing the production of immunoglobulins through the modulation of bacterial antigens.^75,76^ These multifaceted interactions strengthen the host’s defenses against pathogens or other pro-inflammatory stimuli. In particular, probiotics are able to modulate the production of pro-inflammatory factors and enhance anti-inflammatory responses mainly through the regulation of the toll-like receptors (e.g. TLR2 and TLR4) and, in turn, the inactivation of the pro-inflammatory triggers produced by dendritic cells and macrophages.^77^ Concomitantly, probiotics may reduce the protein levels of interleukins and other pro-inflammatory biomarkers, limiting the immune response when unnecessary. Such effects are mediated by the regulation of T-cell differentiation, the activation of T helper cell type 2 (Th2) and the production of anti-inflammatory interleukin (IL)-4 and IL-10.^78^ Overall, probiotics and balanced gut microbiota favor a correct immune state, which prevents chronic inflammation preserving the host’s ability to respond to pathogens and stimuli.

More complex interactions were also described between probiotics and host cells; however, these are not the focus of the present manuscript. What it is important to note is that probiotics establish an intricate interplay within the gut ecosystem and the host, fostering an environment where beneficial microorganisms proliferate and barrier function, mucosal immunity, and immune responses are effective in counteracting harmful pathogens. Therefore, probiotics are widely used for the maintenance of digestive health, to induce immune modulation, metabolic equilibrium, and, more recently, to maintain mental wellness by modulating the so-called “gut-brain axis”.^79^

As regards the fields of application of probiotics, notably, these were first discovered for their pivotal role in promoting digestive health in patients suffering from different gastrointestinal disorders, including irritable bowel diseases, diarrhea, constipation, etc. Several studies also demonstrated that probiotics can modulate gut motility, alleviate abdominal discomfort, and recalibrate stool consistency through their potential to restore gut microbiota imbalances and revert dysbiosis.^80^

Some authors have also proposed probiotic administration as a treatment to limit or mitigate other microbial infections. Accordingly, the regulation of immunoglobulins, cytokines, and immune cells mediated by probiotics contributes to immune resilience and the mitigation of infections.^81^

Furthermore, metabolic disorders emerge as pivotal frontiers where probiotics have been proposed for their potential to mitigate food intolerances and weight gain. Specifically, probiotics actively participate in the degradation of foods by modulating energy extraction from food, promoting fat oxidation, and influencing adipose tissue homeostasis. All these probiotic functions encouraged novel potential strategies to treat metabolic syndrome, obesity and other food-related disorders. In addition, the SCFAs and other metabolites obtained from probiotics further support the adoption of probiotics for the treatment of metabolic disorders.^82^

Recently, neuronal and neurodegenerative disorders have emerged as other fields of application of probiotics. As mentioned above, probiotics actively interact with the gut-brain axis, an intricate bi-directional communication channel that interconnects gut physiology with cognitive functions. As probiotics are effective in maintaining gut eubiosis, researchers have proposed their application to induce mood regulation and improve cognitive functions.^83,84^ Such intricated relationship existing among gut microbiota, probiotics, and cognitive functions seems to be mediated by several factors, including microbial metabolites, neurotransmitters, and neuroinflammatory molecules actively modulated by probiotics.^85^ Certain probiotics ameliorate symptoms of anxiety, depression, and cognitive decline; however, further studies are necessary to unveil the underlying molecular mechanisms through which microorganisms influence neurocognitive functions beyond the gut confines.

Some researchers have reported adverse effects associated with probiotic administration. Although very rare, some probiotic strains may cause systemic infections, particularly in immunocompromised individuals.^12^ Moreover, other findings have demonstrated that some probiotics or postbiotics may lead to allergic reactions in some cases, highlighting how the administration of probiotics should be considered according to the individual response to probiotics or their excipients, as well as the composition of gut microbiota and the clinical conditions of patients.^86^

For these reasons, it is necessary to claim that probiotics are not currently considered as drugs; therefore, they are not supervised by regulatory agencies like the EMA and the FDA. This suggests how standardized guidelines for probiotic use are needed to establish the safety and efficacy of each microorganism in specific clinical settings.

Currently, several probiotics are commercialized alone or in different combinations and brands. Table 1 reports the most widely used probiotics, indicating their primary effects and, eventually, adverse events observed following their administration as reviewed by Zommiti M et al. (2020) and Sanders ME et al. (2010).^60,87^

**Table 1.** Main functions and reported safety concerns of the most used probiotics.

Microorganism	Main Functions	Adverse Events or Safety Concerns ^60,87^	Ref.
*Bacillus clausii (Shouchella clausii)*	Investigated for its role in managing gastrointestinal disorders, especially diarrhea, and supporting gut health	N/A	88
*Bacillus coagulans (Heyndrickxia coagulans)*	Investigated for its potential in promoting gut health and supporting digestive comfort in chronic intestinal disorders like IBS	N/A	89
*Bacillus subtilis (Bacillus inaquosorum)*	Studied for its potential to support gastrointestinal health and immune modulation	N/A	90
***Bifidobacterium adolescentis***	**Investigated for its role in alleviating atopic dermatitis and for its positive effects on cognitive functions**	**β-glucosidase-positive strains convert cycasin in a mutagenic substance**	91
*Bifidobacterium animalis*	Improve lactose digestion and protect the gut from *Enterobacteriaceae* through the production of SCFAs	N/A	92
*Bifidobacterium bifidum*	Researched for its potential in supporting immune function and maintaining gut balance	N/A	23
***Bifidobacterium breve***	**Investigated for its potential in promoting gut health and supporting the immune system**	**Case report of meningitis of an infant with gastroschisis**	93
*Bifidobacterium longum subsp. infantis*	Explored for its potential in promoting gut health and modulating immune responses	N/A	94
*Bifidobacterium animalis subsp. lactis*	Studied for its potential to manage digestive discomfort and immune modulation	N/A	95
*Bifidobacterium longum*	Explored for its role in digestive health and potentially reducing inflammation	N/A	23
***Enterococcus faecium***	**Researched for its potential to support gut health and immune function**	**Potential multiresistance and virulence gene transfer (vancomycin-resistant enterococci)**	96
***Escherichia coli Nissle 1917***	**Used in some formulations to support gut health and balance, especially for the treatment of diarrhea**	**Genotoxic and mutagenic activity due to the presence of the *pks* pathogenetic island producing the mutagen molecule colibactin**	28,97
*Lactobacillus acidophilus*	Known for its potential to promote gut health and supporting digestion	N/A	98
*Lactobacillus amylovorus*	Studies indicate its potential in reducing low-density lipoprotein cholesterol and triglycerides ameliorating metabolic syndrome	N/A	99
*Lacticaseibacillus casei*	Explored for its potential in managing lactose intolerance and promoting gastrointestinal health	N/A	100
*Lactobacillus gasseri*	Ameliorates gastrointestinal functions and favors fat metabolisms with consequent weight loss	N/A	101
*Lactobacillus johnsonii*	Counteracts *H. pylori* infection and inhibits pathogens adhesion in the gut. Recently it showed a protective role for type 1 diabetes	N/A	102
*Lacticaseibacillus paracasei*	Studied for its role in immune modulation and digestive health	N/A	95
*Lactiplantibacillus plantarum (Lactiplantibacillus argentoratensis)*	Investigated for its antimicrobial properties and potential support for gut health	N/A	103
***Limosilactobacillus reuteri***	**Inhibits the growth of Gram-negative (*Escherichia coli*) and Gram-positive harmful bacteria favoring intestinal eubiosis through the production of reuterine e reutericycline. It inhibits also parasites and rotaviruses**	**Increase of fecal calprotectin**	104
*Lacticaseibacillus rhamnosus* GG	Studied for its role in immune system modulation and digestive health. Recent evidence suggests its protective role in cancer	N/A	105
*Ligilactobacillus salivarius*	Studied for its potential to support oral health and gastrointestinal comfort	N/A	106
*Lactococcus lactis subsp. cremoris*	Explored for its potential in immune modulation and digestive health	N/A	107
*Propionibacterium freudenreichii*	Researched for its role in supporting gut health and potentially modulating the immune system	N/A	108
*Saccharomyces boulardii*	A yeast probiotic that may support gastrointestinal health and help manage diarrhea	N/A	109
*Streptococcus salivarius*	Used for the treatment of pharyngotonsillitis as able to produce bacteriocins (salivaricin A2 and salivaricin B) against *Streptococcus pyogenes*	N/A	110
*Streptococcus thermophilus*	Commonly found in fermented dairy products and studied for its potential digestive benefits	N/A	111

## Postbiotics, metabolites, and their potential applications

Postbiotics, also known as metabiotics, have recently attracted growing interest for their potential application as a promising adjuvant strategy in preventing and treating several diseases. According to The International Scientific Association of Probiotics and Prebiotics (ISAPP), a postbiotic is defined as “a preparation of inanimate microorganisms and/or their components that confers a health benefit on the host”.^112^ Therefore, postbiotics refer to non-viable microbial cells and their components, as well as metabolites released after microbial cell lysis or secreted by gut microbes during fermentation processes.^113^ Since they are contained in different fermented products, postbiotics may be naturally assumed with diet. Moreover, postbiotics may be also obtained *in vitro* and commercialized as dietary supplements by using well-defined probiotic bacteria strains processed with specific biotechnological approaches, including biomass production, radiations, high pressure and temperature, and inactivation.^114,115^ Among LAB, *Lacticaseibacillus* and *Bifidobacterium* are the most used for postbiotic production.^116^ Starting from these bacteria strains, it is possible to obtain both postbiotics and probiotic metabolites with beneficial properties, including cell-free supernatant, SCFAs (e.g. acetate, propionate, and butyrate), bacteriocins (e.g. nisin, lactocyclicin, bovicin, and lacticin), non-viable microbial cells or cell envelope components, bacterial lysates, exo- and endo-polysaccharides, vitamins, biosurfactants, cell surface proteins, organic acids, and teichoic acids.^117,118^

Several studies recently showed that postbiotics and probiotic metabolites exert antioxidant, anti-inflammatory, immunomodulatory, and antitumoral activity, highlighting their beneficial effects on host cells.^9,10,20,119,120^ Moreover, it has been reported that postbiotics play a critical role not only in maintaining IEB integrity and functionality but also in inhibiting pathogen infections.^121–123^ Compared to viable bacteria, postbiotics represent a safer alternative strategy for children and immunocompromised patients. Notably, since postbiotics are characterized by non-viable microbial cells, they cannot replicate causing probiotic-related side effects, such as intestinal bacterial overgrowth, presence of viable bacteria in the bloodstream, or activation of genes conferring antibiotic resistance to the host cells.^124–126^ Interestingly, postbiotics or purified probiotic metabolites provide a standardized and consistent dose of bioactive compounds, whereas the effectiveness of probiotics can vary based on several factors, including storage conditions and individual response. Other advantages characterizing postbiotics are represented by higher stability and longer shelf-life than probiotic supplements.^127^ In addition, postbiotics offer benefits without the need for colonization and may be easily incorporated into various products (e.g. functional foods and cosmetics), making them effective for several applications.^128,129^

Currently, the clinical application of postbiotics and probiotic metabolites is still in its infancy and little data are available from *in vivo* studies and clinical trials. Therefore, advanced research focusing on the postbiotics-host interactions is needed to corroborate their employment in clinical and non-clinical settings.

## Probiotic safety

**Figure 2.**
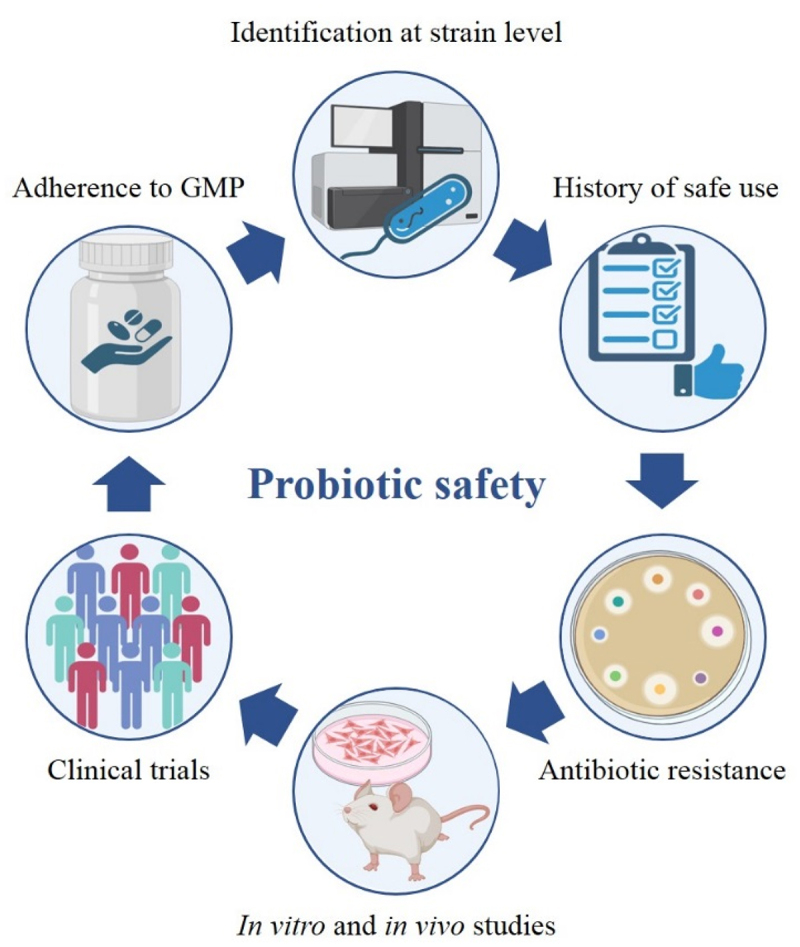
Pipeline for the assessment of probiotic safety.

Over the years, several types of microbes have been proposed as probiotics not only for the maintenance of gut homeostasis but also in clinical settings as an adjuvant treatment. Despite the positive effects of probiotics on host health status have been widely reported, the expanding consumption of probiotic products may also be associated with different adverse events, including gastrointestinal symptoms (e.g. nausea, flatulence, and soft stools), systemic infections, horizontal gene transfer, production of secondary metabolites with genotoxic effects, and excessive immune stimulation in vulnerable subjects.^130,131^ In this field, regulatory requirements for probiotic safety may vary depending on the country and it is difficult to provide globally accepted guidelines (Figure 2).

The first step of the probiotic safety assessment is represented by whole genome sequencing (WGS) of the probiotic candidate to establish bacterial identity at the strain level and retrieve genomic data.^132^ Secondly, a well-documented history of safe use is an important requirement to corroborate probiotic-safe consumption. Notably, the analytic revision of the literature may be a useful benchmark to obtain information on the route of administration, impact on host health, intake levels, as well as any potential side effects. Nevertheless, a history of safe use alone is inadequate for this purpose and a more comprehensive analyses are necessary to confirm probiotic safety.^133,134^ The evaluation of the antibiotic resistance of probiotics is relevant for the use of the candidate strain as a supplement since it could be directly or indirectly transferred to pathogenic bacteria, enhancing their colonization capacity and resistance to treatment. Specifically, the assessment of antibiotic resistance is based on both genotyping and phenotyping approaches, including WGS to detect antibiotic resistance genes and estimation of the minimal inhibitory concentration (MIC) for each clinically relevant antibiotic, respectively.^30,135,136^ Another critical feature that must be considered in probiotic safety is the virulence and toxigenic potential, whose assessment should be based not only on taxonomic identification and evidence of virulence or toxin production but also supported by both short-term and long-term *in vitro* and *in vivo* studies to investigate the mechanisms of action and potential side effects of the strain under investigation.^31,137^ A typical example is represented by the *Escherichia* genus, especially *Escherichia coli* species, which is characterized by several potential pathogenic strains expressing genotoxins and secondary metabolites that have been recently described to have detrimental effects on host cells.^49,138,139^ These strains and other probiotic candidates should be tested for biogenic amines production (e.g. histamine and tyramine) and for mucin metabolism capability, whose excessive degradation is related to chronic diseases like obesity, diabetes, IBD, and CRC.^140,141^ Moreover, large and well-defined clinical trials are another critical component of probiotic safety assessment to collect any adverse events related to probiotic supplements, including nausea, vomiting, fever, constipation, inappetence, and diarrhea.^142–144^ Finally, the production process of probiotic supplements must be conducted in facilities that comply with Good Manufacturing Practice (GMP), ensuring the quality, potency, and purity of these products including the absence of microbial and chemical contaminants.^145,146^

At present, several guidelines and recommendations to establish probiotic safety have been published by International Associations and Agencies including the ISAPP, the Food and Agriculture Organization of the United Nations (FAO), and the World Health Organization (WHO). The first guidelines for the evaluation of probiotic safety in food were produced in 2002 by two joint FAO/WHO working group meetings where two independent reports were obtained. Both reports were then adopted to clearly define “probiotics” and to establish specific recommendations (11 and 6 recommendations established in the Cordoba and London Joint Meeting, respectively) necessary to assess probiotic safety and effects.^147,148^ In brief, these preliminary recommendations include the adoption of a precise definition for probiotics and the necessity to adhere to established guidelines for labeling strains as probiotics. They stressed the importance of establishing a regulatory framework on probiotics, promoting the implementation of good manufacturing practices for probiotic foods, and the establishment of surveillance systems for adverse events associated with probiotics.^147,148^

More recently, the ISAPP elaborated the expert consensus document titled “The International Scientific Association for Probiotics and Prebiotics consensus statement on the scope and appropriate use of the term probiotic”, a document produced at the end of the ISAPP consensus meeting on the appropriate use and scope of the term probiotics which contains also guidelines and recommendations to clearly establish probiotics.^149^ More in detail, this document provided some recommendations including: i) corrections on the old definition of probiotics provided by the FAO/WHO (the new definition is “live microorganisms that, when administered in adequate amounts, confer a health benefit on the host”); ii) considering probiotics only microbial species that have been shown to confer health benefits in properly controlled studies; iii) including more details for products whose label contains the claim “contains probiotics”; iv-v) keep live culture in fermented foods and fecal microbial transplants outside the probiotic frameworks; vi) including as probiotics new bacteria and consortia with adequate evidence of safety and efficacy.^149^ Besides the update provided by the ISAPP in 2014, clear guidelines and recommendations on probiotics safety were already published in 2010 by Sanders ME and collaborators (2010), who actively participated in ISAPP and other relevant Organizations. The authors elaborated a comprehensive manuscript evaluating the effects of probiotics in the context of the potential vulnerability of the consumer or patient, dose and duration of consumption, and both the manner and frequency of administration.^87^ This document was recently updated (2023) still maintaining the main concepts expressed in 2010.^92^ Briefly, these guidelines describe the correct use of probiotics in individuals with immunological defects, critically ill (e.g. hospitalized patients), IBDs, infants, and premature infants. In addition, the authors provided a comprehensive description of microbiological and metabolic issues related to probiotic safety discerning the precise microbial identification, evaluating the colonization properties of probiotics, their antibiotic resistance and the transferability of the genome, the genetic stability/genetic transfer, their pathogenicity and toxigenicity. The documents also described key initiatives in probiotic safety promoted by different consortia, including the European Food Safety Authority (EFSA), the already mentioned FAO/WHO recommendations, the Qualified Presumption of Safety (QPS) document and the PROSAFE European Project.^87,92^ In particular, the QPS is a document introduced by the European Union Scientific Committee on Animal Nutrition in 2002 for the safety assessment of microorganisms used in feed and food applications consisting of four evaluation steps: i) defining the taxonomy of the microbe; ii) gathering sufficient information on its safety in industrial or human environments; iii) excluding pathogenicity; iv) defining its end use. In the case of no safety concerns or if any concerns have been addressed, QPS status may be granted, exempting the microbial strain from further safety assessment. This strategy was first adopted for LAB, including some probiotic species. Compared to the American GRAS system, the QPS is a more flexible tool to assess probiotic safety.^150^

Similarly, the PROSAFE project aims to establish evidence-based safety assessment guidelines for probiotic LAB for human consumption. The project includes five recommendations: i) using molecular methods for taxonomic classification and depositing probiotic strains in public collections; ii) avoiding the development of microbes with abnormal resistance levels to antimicrobials without proper risk assessment; iii) avoiding the use of LAB with known virulence genes, while other properties like bile acid deconjugation were deemed irrelevant for safety assessment; iv) conducting human colonization studies following European guidelines; v) using animal models for safety assessment, specifically rat models of experimental endocarditis.^151^

Overall, the safety and quality assessment of probiotics definitely represents a public concern, which should investigate the risk-to-benefit ratio to guide the medical community on the proper use of probiotics.

## The “curious” case of *Escherichia coli* Nissle 1917

*EcN*, an intestinal Gram-negative bacillus belonging to the B2 phylogenetic group of *Escherichia coli*, was first isolated by Alfred Nissle (serovar O6:K5:H1) in 1917 from the feces of a German soldier which did not develop any gastrointestinal disorder during a severe outbreak of shigellosis in contrast to his comrades. Since then, *EcN* has been formulated as a probiotic supplement, better known as Mutaflor®, and commercialized in Germany and other European countries.^152^ Over the years, *EcN* has been largely employed as an adjuvant for the treatment of numerous intestinal diseases, such as IBD, irritable bowel syndrome (IBS), diarrhea, chronic constipation, and ulcerative colitis (UC), as well as obesity, diabetes, and even gastrointestinal cancers.^153–156^ In particular, *EcN* is characterized by a high colonization and adhesion capabilities to epithelial cells due to the presence of K5 capsule, mobile flagella, and fimbriae. Interestingly, several studies highlighted that the antimicrobial activity of this strain is mainly related to the production of siderophores and cytotoxic necrosis factors.^157,158^ It has also been reported that *EcN* protects IEB functionality, enhancing the expression levels of tight junction proteins and mucin secretion by epithelial cells. Moreover, *EcN* plays a crucial role in immune response modulation, reducing the expression levels of different pro-inflammatory cytokines, including IL-2, Tumor Necrosis Factor (TNF)-α, and Interferon (IFN)-γ.^159–161^ Table 2 summarizes the ongoing and completed clinical trials focusing on the efficacy and safety of the probiotic *EcN* for the treatment of different pathological conditions.

**Table 2.** Ongoing and completed clinical studies on *EcN* (Mutaflor®) deposited in the main registries (https://trialsearch.who.int; https://clinicaltrials.gov).

Study ID	Status	Conditions	Results	Phase	Subjects	Start date	Ref
ACTRN12619000210178	O, NR	CRC	Engineered *EcN* selectively binds tumor tissue and can be used for diagnostic and therapeutic purposes	IV	110	2019	162
NCT03800147	O, NR	*E. coli* infections	Not reported - Unknown status Endpoint: Effects of nutritional fat on the growth and intestinal colonization of *EcN*	N/A	40	2019	N/A
EUCTR2017 -004,531-36-DE	C	CDAD	Not reported - Prematurely ended Endpoint: Reduction of *Clostridium difficile* associated diarrhea in patients treated with *EcN*	II	108	2018	N/A
EUCTR2016 -001,240-19-BG	C	HVs	The intake of *EcN* for 28 days was well tolerated and no safety concerns occurred. After *EcN* cessation a median of 7 days for clearing of the *EcN*.	II	58	2016	N/A
EUCTR2014 -000,936-40-DE	C	T2DM	Low or High dose of *EcN* induce the reduction of HbA1c	III	10	2014	N/A
EUCTR2011 -002,343-99-GB	C	ICU patients	Not reported Endpoint: Reduction of gastric colonization by pathogenic gram negative bacteria in ventilated adult ICU patients treated with *EcN*	II	30	2012	N/A
ACTRN12611000205932	C	IBS	Not reported Endpoint: Improvement of diarrhea and immunological function in patients with IBS treated with *EcN*	N/A	40	2009	N/A
DRKS00000416	C	IBS	Ten weeks of *EcN* administration induce positive effects in IBS patients with gastroenterocolitis or administration of antibiotics	IV	80	2002	163
NCT01013259	C	RC	*EcN*-mediated immunomodulation is not sufficient to achieve clinical efficacy in grass pollen-allergic subjects	II	34	2009	164
NCT02276508	C	UTI	Not reported Endpoint: Evaluate the safety and tolerability of *EcN* in healthy volunteers	I	20	2014	N/A
NCT04608851	O, R	UTI in children	Not reported Endpoint: Evaluation of urinary tract infections recurrence in children treated with *EcN* or placebo	IV	530	2021	N/A
NCT01772615	C	UC	No benefit in the use of *EcN* as an add-on treatment to conventional therapies for active ulcerative colitis	IV	100	2011	165
NCT02706184	C	CRC, GC	Not reported Endpoint: Effect of *EcN* in reducing the duration and intensity of chemotherapy induced diarrhea in gastric and colorectal cancer patients	III	20	2015	N/A
NCT04787276	C	ESLD, HE, LC	*EcN* administration was safe and efficient for the treatment of hepatic encephalopathy. *EcN* reduced ammonia level and inflammation normalizing the gut microbiota composition and improved the cognitive function of patients.	N/A	42	2017	166
NCT05816577	O, NR	Diabetes	Not reported: Endpoint: Evaluation of the safety and properties of a novel, colibactin-knockout *EcN* strain (EcNΔClbP).	I	20	2023	N/A
NCT02726295	C	Constipation	Not reported Endpoint: Effect of *EcN* administration in reducing chronic constipation.	IV	112	2016	N/A
NCT04969679	C	UC	*EcN* was found to be safe and effective in preventing the exacerbation of IBDQ scores and achieving clinical responses and endoscopic remission in patients with mild-to-moderate UC.	IV	134	2018	167
NCT02802059	C	Infections	Short-term administration of *EcN* reduces infection in newborns, however, the long-term effect of *EcN* gut colonization was not demonstrated.	III	567	2015	168
NCT01469273	O	Infantile diarrhea	Not reported Endpoint: Evaluation of the efficacy and tolerance of a suspension of *EcN* on prophylaxis against gastrointestinal infections in newborn and infants.	IV	198	2011	N/A
NCT02953171	O	IBS	Not reported Endpoint: Effects of lacto-fermented sauerkraut or *EcN* in the treatment of IBS patients.	N/A	140	2016	N/A
NCT05377112	C	EH	Not reported Endpoint: Evaluation of the safety, tolerability, and oxalate lowering in subjects with a history of gastric bypass surgery or short-bowel syndrome treated *EcN*.	I	11	2022	N/A

*Abbreviations*: **C**, Completed; **CDAD**, *Clostridium difficile* Associated Diarrhea; **CRC**, Colorectal Cancer; **EH**, Enteric Hyperoxaluria; **ESLD**, End-Stage Liver Disease; **GC**, Gastric Cancer; **HE**, Hepatic Encephalopathy; **HVs**, Healthy Volunteers; **IBS**, Irritable Bowel Syndrome; **ICU**, Intensive Care Unit; **LC**, Liver Cirrhosis; **N/A**, Not Applicable; **NR**, Not yet Recruiting; **O**, Ongoing; **R**, Recruiting; **RC**, Rhinoconjunctivitis; **T2DM**, Diabetes Mellitus Type II; **UC**, Ulcerative Colitis; **UTI**, Urinary Tract Infections.

Among the reported clinical trials, the NCT05816577 study entitled “Safety and Viability of an *E. Coli* Nissle Colibactin Knockout in Healthy Volunteers” investigated the safety and properties of a colibactin-knockout *EcN* strain (*EcN ΔClbP*) through a randomized, controlled intervention trial in healthy subjects (https://clinicaltrials.gov). In this regard, previous studies demonstrated that *EcN* genome harbors the *pks* (polyketide synthase) pathogenicity island encoding for a hybrid peptide-polyketide genotoxin, known as colibactin, for which several reports showed a critical role in the pathogenesis of CRC due to the induction of DNA DSBs and interstrand cross-links (ICLs) responsible for chromosomal abnormalities and gene mutations.^169,170^ Recently, Huber AR and colleagues (2024) observed that intestinal organoids co-cultured with a colibactin-producing *EcN* presented mutational patterns similar to those observed in CRC. In addition, the authors demonstrated that colibactin-mediated mutations are responsible for the development of CRC diagnosed at a younger age.^171^ These findings are in line with those obtained by other research groups independently. Pleguezuelos-Manzano C et al. (2020), tested human intestinal organoids with both genotoxic *pks*^+^ *EcN* and *pks*^−^ mutant *EcN* showing a unique mutational signature in organoids treated with colibactin-producing bacteria. This colibactin-related signature was also found in a subset of CRC genomes, suggesting a direct link between exposure to colibactin-producing bacteria and CRC mutagenesis.^172^ The genotoxic effects of *EcN* were also demonstrated by treating primary murine colon epithelial organoids with short-term exposure to colibactin-producing *EcN*. Specifically, the authors observed that organoids infected with *pks*^+^ *EcN* presented CRC-like phenotype including increased proliferation, Wnt-independence, and impaired cell differentiation. NGS experiments also revealed an elevated mutational burden, chromosomal aberrations, and epigenetic alterations affecting the p53-signaling pathway, including the dysregulation of miR-34a.^173^

These data suggest how the precise characterization of *EcN* safety is mandatory to classify it as a probiotic compared to other *E. coli* strains. In this regard, a recent study tested the safety and kinetics of *EcN* both in humans and nonhuman primates demonstrating the safety of *EcN* with a probiotic clearance obtained after 1 week of treatment cessation in humans and a longer period in cynomolgus monkeys.^174^ In addition, wet and *in silico* metagenomic studies confirmed the presence of several gene products associated with the production of potentially harmful secondary metabolites, including 40 associated with colibactin, 10 with salmochelin, 30 with aerobactin, and nine with yersiniabactin and enterobactin. The same results were obtained by comparing *EcN* with other *E. coli* probiotic strains (e.g. CEC15) where the presence of colibactin-producing genes was not found.^175,176^ Other studies compared the genomics and metabolomics profile of nonpathogenic *EcN* with other pathogenic *E. coli* strains revealing strong genetic similarities between *EcN* and UPEC strain CFT073; however, this latter possesses several virulence factors, like the toxin α-hemolysin and type 1, P, and F1C fimbrial adhesins, which lack in the probiotic strain *EcN* (serotype O6:K5:H1) that is thus considered nonpathogenic.^177,178^

Although *EcN* has been administered to patients for over a century, the identification of colibactin raises a safety issue that must be further investigated to clarify the pathogenicity and/or probiotic effect of the *EcN* strain.

### Colibactin biosynthesis and genotoxic activity

**Figure 3.**
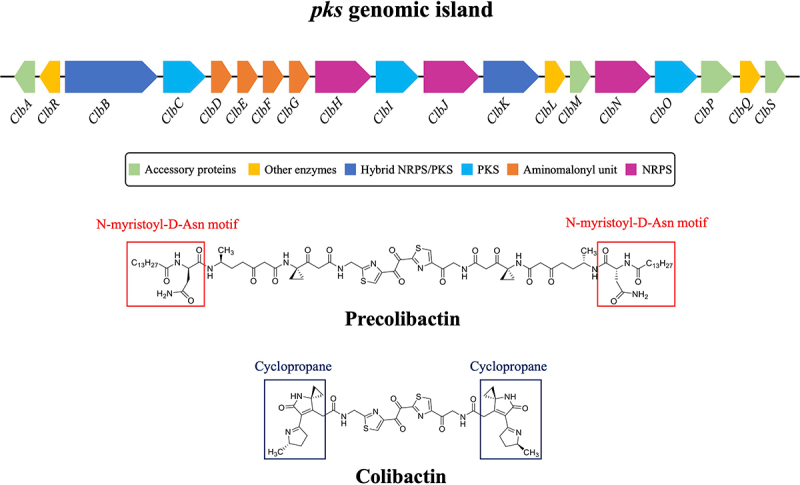
Structure of *pks* genomic island (*ClbA* – *ClbS*) and colibactin biosynthesis.

Colibactin, a secondary metabolite belonging to the heterogeneous bacterial toxin family of cyclomodulins, is synthesized by a genomic island termed *pks*. Interestingly, this pathogenic island has been identified in different bacteria of the *Enterobacteriaceae* family, especially *Klebsiella pneumoniae* and *Escherichia coli* strains of the B2 phylogenetic group, which may be found as commensal microbes in the gut microbiota and in a variety of infections as opportunistic pathogens.^179,180^ The *pks* island (54-kb) consists of a gene cluster of 19 genes (*ClbA* – *ClbS*), which have a functional role in colibactin biosynthesis. Specifically, *ClbH*, *ClbJ*, and *ClbN* encode for various NRPS (Non-Ribosomal Peptide Synthetase) enzymes, while *ClbC*, *ClbI*, and *ClbO* encode for PKS enzymes (Figure 3).^181,182^ The remaining members of this cluster are involved in the encoding of hybrid NRPS-PKS enzymes (*ClbB* and *ClbK*), proteins responsible for the synthesis and transfer of amminomalonyl unit (*ClbD – ClbG*), accessory proteins (*ClbA*, *ClbM*, *ClbP*, and *ClbS*), and other enzymes (*ClbL*, *ClbQ*, and *ClbR*) (Figure 3).^181,182^ The colibactin biosynthetic pathway is regulated by the transcriptional activator ClbR and the phosphopantetheinyl-transferase (PPTase) ClbA. Following transcriptional activation, the hybrid NRPS-PKS enzymes catalyze the production of the intermediate pre-colibactin, which is characterized by a structural motif at the N-terminus (N-myristoyl-D-Asn motif).^183,184^ Precolibactin is then translocated into the periplasm and converted into the genotoxin colibactin via the transporter ClbM and the peptidase ClbP, respectively. Notably, the production of the mature colibactin is due to the removal of the N-terminus structural motif, which induces cyclization of the linear intermediate. The bioactive molecule results in two symmetrical subunits containing cyclopropane, which show a high affinity for binding adenine residues on DNA (Figure 3).^183,184^

As widely reported in the literature, colibactin-producing bacteria showed detrimental effects on host cells inducing DNA DSBs and chromosome aberrations.^173,185,186^ Among the most frequently observed structural aberrations, colibactin induces single nucleotide variants (SNV), insertion and deletion (indel) mutations, and rearrangement breakpoints. In a model of epithelial colon cells, Iftekhar A and colleagues (2021) identified multiple copy number variants (CNV), including whole chromosomes or chromosome arm loss.^173^ Other groups identified a colibactin mutational signature consisting of the increase of *T* > N substitutions at ATA, ATT, and TTT trinucleotides repeats, the increase of small indels at A:T homopolymers, and the enrichment of structural variant breakpoints affecting the AAWWTT motif.^172,187^ As a general mechanism, colibactin acts as a DNA alkylating agent leading to DNA ICLs and DSBs. These breaks trigger cell-cycle arrest and ultimately promote the neoplastic transformation of cells.^188^

Moreover, a higher abundance of *pks*^*+*^ *Escherichia coli* has been detected in CRC patients compared to healthy controls, highlighting that colibactin overexpression could play a critical role in tumor development and invasiveness.^49,172,187^ Although previous studies have provided deep insights into the biosynthesis and activity of colibactin, further investigations are needed to clarify the genotoxic potential of bacterial strains producing this metabolite.

### The potential genotoxic effect of EcN

Over the years, the probiotic *EcN* has been widely employed as an adjuvant for the management and treatment of various intestinal disorders, especially IBD and UC.^189–192^ Despite a long history of safe use as a beneficial bacterial strain with no obvious side effects, the genomic identification of the *pks* locus encoding colibactin has recently raised a safety concern since this genotoxin could play a pivotal role in CRC development inducing DNA damage and gene mutations.^139,193,194^ In this field, a number of recent studies investigated the *EcN* biosafety to assess any genotoxic effect (Table 3).

**Table 3.** Recent *in vitro* and *in vivo* studies evaluating the probiotic and genotoxic potential of the *EcN* strain.

Model	Study Protocol	*EcN* Effects	Ref.
*In vitro* (Comet assay on Caco-2 cells and Ames test on *EcN*)	Treatment with viable *EcN* (multiplicity of infection of 1:500) for 90 min at 37°C	**P:***EcN* exerts probiotic effects reducing the mutagenic activity of NQO, H_2_O_2_, and B[a]P	195
*- In vitro (*Comet assay on rat cells and Ames test on viable *EcN* and supernatant) *In vivo (*Comet assay and genotoxicity test on SPF rats)	*- In vitro* test: Treatment with *EcN* supernatant obtained from 1–2·10^11^ CFU/mL diluted from 1:2 to 1:100 *- In vivo* test: Treatment with live *EcN* with a daily dose of 1.1·10^7^ CFU/kg	**P:***EcN* does not have mutagenic, genotoxic, or DNA-damaging activity in both *in vivo* and *in vitro* tests	196
*In vitro* (Human Intestinal Organoids – HIO)	- 24h treatment using 10^3^ CFU of *EcN* microinjected into the HIO lumen - 24h treatment performing co-infection with 10^3^ CFU of *EcN*, EHEC and UPEC	**P:***EcN* improves IEB function, host defense, and E-cadherin levels in HIO. *EcN* also reduces ROS, apoptosis, EHEC and UPEC strains growth	197
*- In vitro* (rat IEC-6 cells) - I*n vivo* (Wistar rats and SCID mice)	*- In vitro*: Treatment with infection dose of 20 or 100 bacteria per cell *- In vivo*: daily oral gavage of 10^9^ CFU of *EcN* dissolved in PBS for 45 days	**P:***EcN* induces the reduction of colon inflammation, colitis, MPO activity and IL-1β levels **G:** *EcN* increases DNA DSBs, chromosomal abnormalities, and IL-10 levels	198
*In vitro* (Caco-2 and HT-29 cells)	Treatment with 5 μg/ml of outer membrane vesicles (OMV) from *EcN* for 168 h	**P:***EcN* OMV reduces HT-29 cell proliferation and oxidative stress. No alteration of cell viability **G:** *EcN* OMV induces DNA DSBs	199
*- In vitro* (HeLa cells) - *In vivo* (C57BL/6 mice)	- *In vitro*: Treatment with 5 x 10^8^ CFU/ml of *EcN* for 4h - *In vivo*: Treatment with 10^9^ CFU of *EcN* for 4 days	**P:***EcN* showed antibacterial activity toward *S. Typhimurium* in vivo G: production of colibactin and increase of DNA damages	200
*- In vitro* (HeLa and CHO cells) *- In vivo* (SPF and BALB/c mice)	*- In vitro*: Cells were treated with a dose of multiplicity of infection of 400 bacteria per cell for 4h *- In vivo*: SPF mice were treated with 10^10^ CFU/ml of *EcN* for 6h; BALB/c mice were treated by intragastric gavage with 10^8^ *EcN* suspended in PBS and sacrificed after 7 days	G: *EcN* induces colibactin synthesis, DNA ICLs, gene mutation frequency, abnormal mitosis, and DNA damage	28

*Abbreviations*: **B[a]P**, Benzo[a]pyrene; **DNA DSBs**, DNA Double-Strand Breaks; **DNA ICLs**, Interstrand Cross-Links; **EHEC strains**, Enterohemorrhagic *E. coli* strains; **G**, Genotoxic; **H**_**2**_**O**_**2**,_ Hydrogen peroxide; **HIOs**, Intestinal Human Organoids; **IEB**, Intestinal Epithelial Barrier; **IL-10**, Interleukin-10; **IL-1β**, Interleukin-1β; **MPO**, Myeloperoxidase; **NQO**, 4-nitroquinoline-1-oxide; **OMV**, Outer Membrane Vesicles; **P**, Probiotic; **ROS**, Reactive Oxygen Species; **SCID**, Severe Compromised Immunodeficient; **SPF**, Specific-Pathogen-Free; **UPEC**, Uropathogenic *E. coli* strains.

Janosch D et al. (2019) performed *in vitro* tests (Comet Assay and Ames test) to evaluate the activity of *EcN* strain against standard mutagens NQO (4-nitroquinoline-1-oxide), H_2_O_2_ (hydrogen peroxide), and BaP (benzo[a]pyrene). Of note, preincubation with *EcN* preparation significantly reduced the genotoxic activity of such mutagenic compounds, indicating that the probiotic *EcN* could play a pivotal role in maintaining the health status of the host.^195^ Similarly, Dubbert S and colleagues (2020) reported that *EcN* cell-free supernatant showed no mutagenic activity. These results were further corroborated on animal models by testing the genotoxicity of the *EcN* product (1·10^11^ colony-forming unit (CFU)/mL) orally administered for 28 consecutive days. Notably, no difference was detected when analyzing the intestinal tissue of treated and control rats, suggesting that *EcN* did not exert any genotoxic activity.^196^ However, it is important to note that the standard assays used by Dubbert S and colleagues were considered inappropriate by Nougayrède JP and colleagues. Indeed, they used an Ames test in which *Salmonella enterica* serovar Typhimurium reporter bacteria were exposed to *EcN*, and then *Salmonella* growth was expected upon mutagenesis. Moreover, *Salmonella* is readily killed by the siderophores/microcins produced by *EcN*; thus, the absence of growth of the reporter bacteria was incorrectly interpreted as an absence of the effect of colibactin. In addition, Dubbert S et al. used a standard *in vitro* Comet assay to analyze the exposed intestinal epithelial cells of rats; the Comet assay detected a variety of DNA lesions through electrophoresis of broken DNA but could not detect DNA cross-links that inhibit DNA electrophoretic mobility. Thus, the assays they used cannot detect colibactin-associated mutagenic damage.^28^ In 2020, another study explored the pathogenic potential of *EcN* compared to enterohemorrhagic (EHEC) and uropathogenic (UPEC) *Escherichia coli* strains.^197^ Using human intestinal organoids, the authors observed that the *EcN* treatment did not affect the IEB structure and function, whereas EHEC and UPEC strains destroyed the intestinal tissues. Moreover, organoid co-cultures revealed that *EcN* guaranteed the IEB integrity, enhancing immune defenses and inhibiting the growth of pathogenic bacteria.^197^

Although the aforementioned studies indicated that *EcN* may be considered a safe and efficient probiotic strain for host gastrointestinal health, heterogeneous findings are reported in the literature regarding the potential genotoxic effect of *pks*^*+*^ *EcN* on host cells (Table 3). In this regard, Olier M and colleagues (2012) treated the IEC-6 cells with a non-genotoxic mutant of *EcN* (*ΔclbA*), observing no detrimental effect compared to wild-type *EcN*, which induced DNA DSBs to intestinal cells. Simultaneously, oral administration of the *EcN ΔclbA* (10^9^ CFU/day) to animal models of colitis revealed that the isogenic mutant did not protect the colon, while the supplementation of wild-type *EcN* decreased the severity of the inflammatory status, suggesting that the probiotic effect of *EcN* is strictly associated to its genotoxic activity.^198^ Interestingly, Cañas MA et al. (2016) also evaluated the potential genotoxic/cytotoxic effects of outer membrane vesicles (OMVs) derived from the probiotic *EcN*. Notably, the authors demonstrated *in vitro* that *EcN* did not affect cell viability of the intestinal epithelial cell lines; however, the exposure to OMVs (5 μg/mL for 48 h) induced DNA DBSs in HT-29 cells, suggesting that the synthesis of colibactin could be implicated in the *EcN* genotoxic effect.^199^ Another study focused the attention on the beneficial effects of *EcN*-derived OMVs demonstrating a potential modulating effect on gut and liver metabolism and a better control of obesity and diabetes in mice models.^200^ Similarly, Han L et al. (2024) recently tested the efficacy of *EcN*-OMVs in reducing intestinal inflammation by developing an innovative delivery system using aldehyde-silica microspheres. The engineered vesicles showed better stability to gastric juice both *in vitro* and *in vivo* as well as increased anti-inflammatory effects reducing the expression of TNF-α and IL-1β, and increasing the expression of zonula occludens-1.^201^

The close interaction between the probiotic activity and the genotoxic potential of *EcN* was also evaluated by Massip C et al. (2019), focusing on ClbP, an essential enzyme for the synthesis of mature colibactin. Interestingly, the authors reported that the antimicrobial activity was significantly reduced in *ΔClbP* mutant compared to *ΔclbA* mutant and wild-type *EcN*, highlighting that the *pks* genomic island could be responsible not only for the genotoxic activity of *EcN*, but also for its probiotic function.^202^ Similarly, the genotoxic and mutagenic properties of *EcN* were further confirmed by Nougayrède JP and colleagues (2021). Specifically, the authors noted that the infection of epithelial cells with *EcN* wild type caused the synthesis of colibactin leading to DNA ICLs and gene mutations, while no DNA damages were observed for the *ΔclbA* and *ΔClbP* mutants. Moreover, the genotoxic activity of colibactin-producing *EcN* was also demonstrated through the analyses of colon tissue samples derived from mice treated with *EcN* suspensions (10^8^ CFU/mL).^28^

Overall, the conflicting results of the above-mentioned studies highlight a serious safety concern that must not be ignored. Therefore, deeper investigations are mandatory to provide novel insights into the long-term risk-to-benefit ratio of the probiotic *EcN*. Despite the safety concerns here reported, at present, there are no specific recommendations for the administration of *EcN*. Some authors have demonstrated *in vivo* that the administration of *EcN* to immunocompromised mice with altered gut microbiota is not recommended as they can develop severe adverse events, including septic episodes and impaired IEB functions. Since *EcN* is also used to reduce the incidence of necrotizing enterocolitis and death in very low-birth-weight preterm infants, it should be noted that a case of severe sepsis in a preterm infant was observed after the administration of *EcN*.^203^ Interestingly, according to the European Crohn’s and Colitis Organization (ECCO), *EcN* is the only recommended probiotic for the treatment of ulcerative colitis as demonstrated by multiple clinical studies.^163,192^ Beyond these isolated recommendations, to date there are no consensus documents on the correct use of *EcN*, therefore, efforts in this direction are necessary.

## Conclusions

As widely reported in the literature, gut microbiota eubiosis and host health status are strictly interconnected. However, any alteration of such equilibrium may lead to dysbiotic microbiota and a higher risk of acute and chronic gastrointestinal disorders associated with the overgrowth of pathogens and the production of toxic metabolites. In this context, both probiotics and postbiotics have been largely employed as adjuvant therapeutic strategies to restore the composition of the gut microbiota and ameliorate host health conditions by exerting anti-inflammatory and immunomodulatory effects. *EcN* is a typical example of probiotic mainly used for the treatment of gastrointestinal disorders like IBD and UC. Despite a well-documented history of safe use, the identification of the *pks* genomic island encoding the genotoxin colibactin suspected to induce DNA damage and CRC has raised a safety concern regarding the use of *EcN*. In this field, a number of recent studies focused on *EcN* biosafety, investigating its genotoxic potential both *in vitro* and *in vivo*. Overall, these studies have all indicated that *EcN* may be considered a safe and efficient probiotic strain for host gastrointestinal health; however, some reports highlighted the genotoxic effect of colibactin produced by *pks*^+^ *EcN* on host cells able to cause DNA damage and genetic mutations.

Therefore, the exact boundary between the probiotic and pathogenicity activity of the *EcN* strain has not been fully deciphered yet due to the conflicting findings of studies reported in the present review article. Therefore, since the *EcN* genotoxic potential remains an open question of paramount importance, further studies are mandatory to establish its safety and define the optimal use of this probiotic as an adjuvant therapy for gastrointestinal disorders.

## Data Availability

The data reported in the present manuscript are available at https://pubmed.ncbi.nlm.nih.gov. (accessed on 26 October 2023) and at https://clinicaltrials.gov (accessed on 20 November 2023).
